# A novel melittin nano-liposome exerted excellent anti-hepatocellular carcinoma efficacy with better biological safety

**DOI:** 10.1186/s13045-017-0442-y

**Published:** 2017-03-20

**Authors:** Jie Mao, Shujun Liu, Min Ai, Zhuo Wang, Duowei Wang, Xianjing Li, Kaiyong Hu, Xinghua Gao, Yong Yang

**Affiliations:** 1 State Key Laboratory of Natural Medicines, Jiangsu Key Laboratory of Drug Discovery for Metabolic Disease, Center for New Drug Safety Evaluation and Research, China Pharmaceutical University, Nanjing, 211198 China; 2 The Second Hospital of Nanjing, Nanjing, 320100 China; 3 School of Pharmacy, Nanjing University of Chinese Medicine, Nanjing, 210023 China; 4 Institute of Pharmaceutical Science, China Pharmaceutical University, No 639, Longmian Rd., Nanjing, 211198 China

**Keywords:** Bee venom, Melittin nano-liposomes, Anti-tumor activity, Biological safety

## Abstract

Melittin is the main effective component of bee venom and has extensive biological functions; however, serious side effects have restricted its clinical application. Preclinical and clinical studies showed that the main adverse events were allergic reaction and pain at the administration site. To decrease the toxicity, we prepared melittin nano-liposomes by encapsulating melittin with poloxamer 188 and explored the inhibitory activities on liver cancer together with biological safety. Here, we showed that melittin nano-liposomes significantly inhibited the survival of hepatocellular carcinoma (HCC) cells in vitro and prominently suppressed the growth of subcutaneous and orthotopic HCC transplantation tumors in vivo. It was important that it induced less inflammation and allergy in mice compared with melittin. Overall, melittin nano-liposomes would have a better application in HCC therapy due to its significant anti-tumor activity and better biological safety.

**Electronic supplementary material:**

The online version of this article (doi:10.1186/s13045-017-0442-y) contains supplementary material, which is available to authorized users.

## Letter to the editor

Melittin is the main effective component of bee venom and has extensive biological functions in vivo, including anti-cancer property. To evaluate the anti-cancer activity of melittin on hepatocellular carcinoma (HCC), a clinical trial containing 40 HCC patients was conducted in Yancheng Second People’s Hospital (Yancheng, China). Patients with melittin treatment showed partial remission (PR) (*n* = 4, 10%), stable disease (SD) (*n* = 24, 60%), and progressive disease (PD) (*n* = 12, 30%), and the disease control rate (CR + PR + SD) was 70%. Toxicity was also assessable in the 40 patients. The most common adverse events were pain at the administration site and skin itch, which disappeared after melittin withdrawal (32 grade 0, 6 grade I, and 2 grade II). This results together with other preclinical studies of melittin indicated that it exerted a significant anti-HCC activity, while serious side effects have restricted the clinical application of melittin in cancer therapy [1–4]. To resolve these problems, melittin was modified with 2% poloxamer 188 and melittin nano-liposomes were prepared (Patent number: CN 101391098 A).

**Fig. 1:**
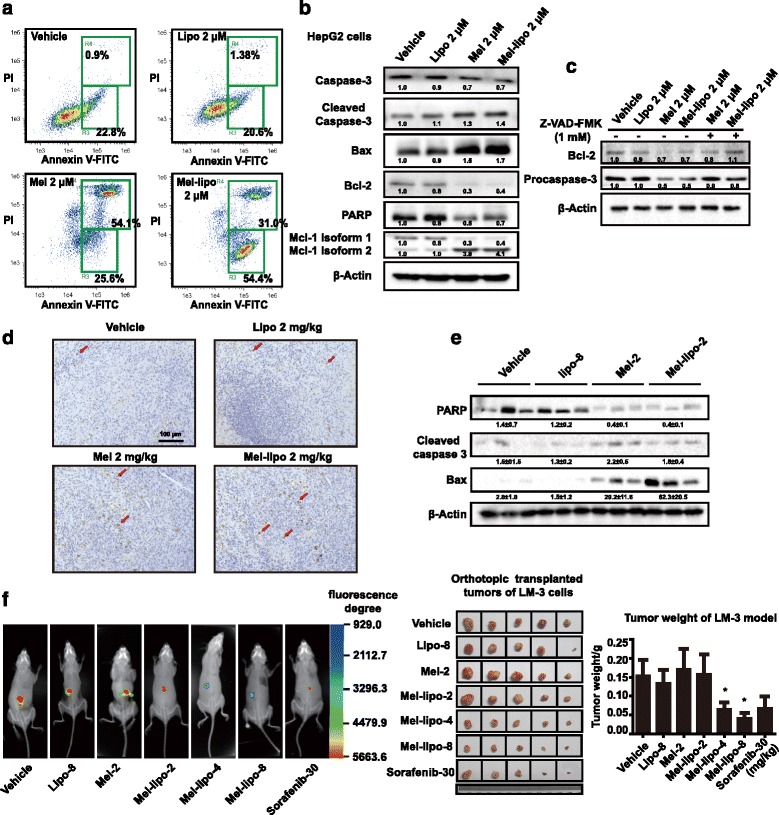
Melittin nano-liposomes induced apoptosis in hepatic carcinoma cells in vitro and vivo and inhibit hepatocellular carcinoma in LM-3 xenograft tumor model. **a** HepG2 cells were cultured with vehicle, blank liposomes (2 μM), melittin (2 μM), or melittin nano-liposomes (2 μM) for 24 h, stained with annexin V-FITC and PI, and analyzed by flow cytometry. **b** Western blot analysis of apoptosis-related proteins after HepG2 cells were treated with vehicle, liposomes (2 μM), melittin (2 μM), or melittin nano-liposomes (2 μM) for 24 h. **c** HepG2 cells were pretreated with or without the caspase inhibitor Z-VAD-FMK for 6 h and then treated with vehicle, liposomes (2 μM), melittin (2 μM), or melittin nano-liposomes (2 μM) for 24 h. Western blot analysis of Bcl-2 and procaspase-3 was carried out subsequently. **d** TUNEL assay for detecting apoptosis in tumor tissues of the hepatocellular carcinoma Hepa 1-6 orthotopic tumor model. **e** Western blot analysis of apoptosis-related proteins of Hepa 1-6 tumor tissues. **f** Live imaging photos and the fluorescence degree of vehicle, blank liposomes (8 mg/kg), melittin (2 mg/kg), melittin nano-liposomes (2, 4, and 8 mg/kg), and sorafenib (30 mg/kg) treated groups in the LM-3 orthotopic implanted tumor model. The data are presented as the mean ± SEM. Statistical significance was calculated using Student’s *t* test (*p ≤ 0.05)

The anti-tumor activity of melittin nano-liposomes was investigated both in vitro and in vivo models (Additional file 1: Materials and methods). In the current study, we found that five hepatic carcinoma cell lines used (Bel-7402, BMMC-7721, HepG2, LM-3, and Hepa 1-6 cells) were sensitive to melittin nano-liposomes, and the IC_50_ value was close to melittin, ranging from 1.44 to 2.1 μM (Additional file 2: Table S1). Melittin and melittin nano-liposomes could remarkably induce cell apoptosis compared with vehicle or blank liposomes (Fig. 1a and Additional file 3: Fig. S1). Western blot analysis showed that melittin nano-liposomes (2 μM) increased the expression level of pro-apoptotic proteins, such as Bax and cleaved caspase-3, and decreased anti-apoptotic proteins, including Bcl-2 and PARP, in HepG2 cells compared with the vehicle or blank liposome group (Fig. 1b). The apoptosis process could be partly reversed by the caspase inhibitor Z-VAD-FMK (Fig. 1c). To assess the apoptosis induced by melittin nano-liposomes in vivo, a liver orthotopic xenograft tumor model of Hepa 1-6 cell was established. TUNEL assay revealed that tumor tissues of the melittin nano-liposome (2 mg/kg) group owned a larger proportion of apoptotic cells than the control or blank liposome groups, and the expression levels of apoptosis-regulated proteins in tumor tissue were also detected (Fig. 1d–f). Melittin nano-liposomes also showed significant inhibition of hepatocellular carcinoma growth in two nude mouse models, including the HepG2 cell subcutaneous xenograft model and LM-3-GFP cell orthotopic xenograft model (Fig. 1g and Additional file 4: Fig. S2).

**Fig. 2:**
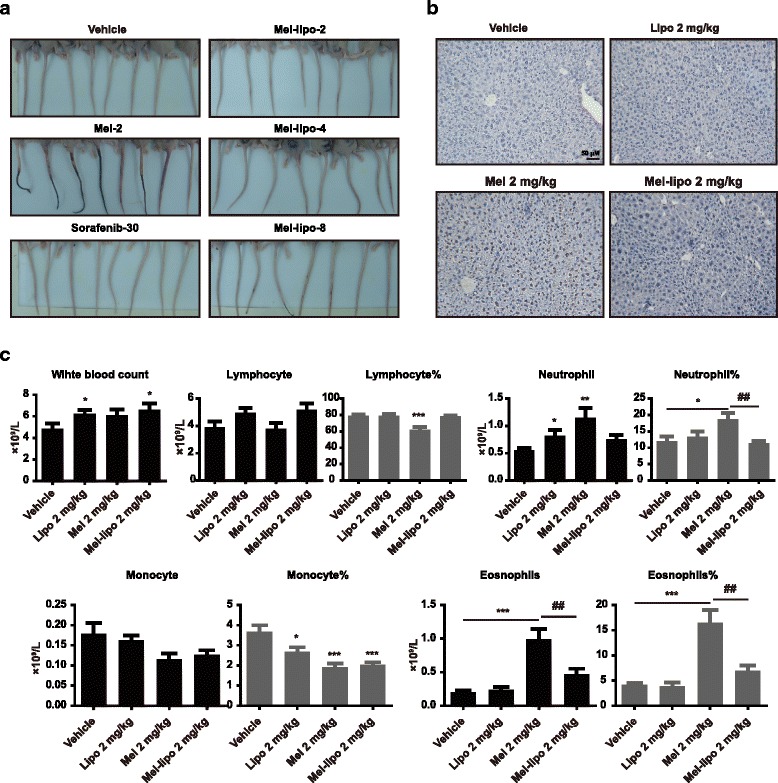
Melittin nano-liposomes showed reduced toxicity in vivo. **a** Photos of the tails of each group at the end point of treatment. These mice were from the LM-3 model. **b** TUNEL assay for detecting apoptosis in the liver tissue after mice (*n* = 10) were treated with vehicle, blank liposomes (8 mg/kg), melittin (2 mg/kg), and melittin nano-liposomes (2 mg/kg) for 2 weeks. **c** Peripheral blood was collected from the ICR mice and analyzed by automated hematology analyzer

To evaluate the toxicity of melittin and melittin nano-liposomes, we primarily compared the injury severity of the mouse tails in the LM-3 model where the drugs were administered. The tails of the melittin group showed severe tissue swelling and necrosis while the tails of the melittin nano-liposome group were injured less, even at a high dose of 8 mg/kg (Fig. 2a). To further compare the biological safety of melittin and melittin nano-liposomes, the drugs were intraperitoneally administered to ICR mice for 2 weeks. TUNEL assay revealed that melittin caused slight apoptosis in hepatocytes while melittin nano-liposomes showed lower toxicity to the liver tissue (Fig. 2b). Meanwhile, melittin caused a decrease of lymphocyte percentage and an increase of neutrophils in both peripheral blood and spleen, suggesting that melittin induced an inflammatory response in vivo; however, melittin nano-liposomes showed similar lymphocyte and neutrophil percentages as the control groups. Melittin induced an allergic reaction with significantly increased eosinophils and eosinophil percentage in blood, while melittin nano-liposomes effectively prevented anaphylaxis in the mice. Liposomes and melittin nano-liposomes also caused an increase of splenic B lymphocytes. (Fig. 2c and Additional file 6: Fig. S3).

In summary, our results revealed that melittin significantly delayed HCC development with certain side effects in clinic trial. Novel melittin nano-liposomes showed outstanding anti-HCC potency in vitro and in vivo with a decreased toxicity. The results potentially have clinical implications for melittin nano-liposomes as a promising new drug for HCC therapy.

## Additional files

Additional file 1: Materials and methods. (DOCX 24 kb)
Additional file 2: Figure S1. The effects of melittin nano-liposomes on the proliferation and apoptosis of tumor cells. (a) Proliferation inhibiting rates of melittin and melittin nano-liposomes on various HCC cells lines including LM-3, Bel-7402, SMMC-7721, HepG2, and L02 cells. (b) Cell nucleus staining by DAPI to observe the apoptosis of Bel-7402 and SMMC-7721 cells after treated with vehicle, blank liposomes (1 μM), melittin (1 μM), or melittin nano-liposomes (1 μM) for 24 h. (c) Fluorescence staining of Annexin V-FITC and PI to detect the apoptosis of HepG2 cells after treatment with melittin and Melittin nano-liposomes for 24 h and observed by fluorescence microscope. HepG2 cells were pretreated with Z-VAD-FMK for 6 hours, and melittin and Melittin nano-liposomes were subsequently administered at a concentration of 2 μM. (PDF 848 kb)
Additional file 3: Figure S2. Photo of HepG2 tumors of vehicle, blank liposomes (8 mg/kg), melittin (2 mg/kg), melittin nano-liposomes (2, 4, and 8 mg/kg), and sorafenib (30 mg/kg) treated groups in the HepG2 subcutaneous transplanted tumor model. The data are presented as the mean ± SEM. Statistical significance was calculated using Student’s *t* test (***p* ≤ 0.01; ****p* ≤ 0.001). (PDF 1070 kb)
Additional file 4: Figure S3. Flow cytometry analysis of splenic immune cells including splenic T lymphocytes, neutrophil and B lymphocytes. Spleens were stripped and grinded into cell suspension after mice were treated with vehicle, blank liposomes (8 mg/kg), melittin (2 mg/kg) and melittin nano-liposomes (2 mg/kg) for two weeks. (PDF 730 kb)

## Abbreviations

HCC: Hepatocellular carcinoma
PD: Progressive disease
PR: Partial remission
SD: Stable disease
