# Correction to: A novel melittin nano-liposome exerted excellent anti-hepatocellular carcinoma efficacy with better biological safety

**DOI:** 10.1186/s13045-022-01277-5

**Published:** 2022-09-09

**Authors:** Jie Mao, Shujun Liu, Min Ai, Zhuo Wang, Duowei Wang, Xianjing Li, Kaiyong Hu, Xinghua Gao, Yong Yang

**Affiliations:** 1 State Key Laboratory of Natural Medicines, Jiangsu Key Laboratory of Drug Discovery for Metabolic Disease, Center for New Drug Safety Evaluation and Research, China Pharmaceutical University, Nanjing, 211198 China; 2 The Second Hospital of Nanjing, Nanjing, 320100 China; 3 School of Pharmacy, Nanjing University of Chinese Medicine, Nanjing, 210023 China; 4 Institute of Pharmaceutical Science, China Pharmaceutical University, No 639, Longmian Rd., Nanjing, 211198 China

## Correction to: Journal of Hematology & Oncology (2017) 10:71 10.1186/s13045-017-0442-y

The original article [1] contained an error in Fig. 1 which has since been corrected.

## References

[1] Mao J (2017). A novel melittin nano-liposome exerted excellent anti-hepatocellular carcinoma efficacy with better biological safety. J Hematol Oncol.

