# Supplementary material for: A novel melittin nano-liposome exerted excellent anti-hepatocellular carcinoma efficacy with better biological safety
Source: J Hematol Oncol. 2017 Mar 20;10:71. doi: 10.1186/s13045-017-0442-y (PMC5359812; doi:10.1186/s13045-017-0442-y)
Supplement: Additional file 1: — Materials and methods. (DOCX 24 kb) [file 13045_2017_442_MOESM1_ESM.docx]

**Materials and Methods**

*Cell lines and animals*

A mouse hepatic carcinoma cell line (Hepa 1-6 cells) and human hepatic carcinoma cell lines (Bel-7402, BMMC-7721, L02 and HepG2 cells) were purchased from the Shanghai Cell Collection (Chinese Academy of Sciences, Shanghai, China). The LM-3-GFP cell line (LM-3 cells transfected with green fluorescent protein) was purchased from Nanjing Gulou Hospital (Nanjing, China). All the cell lines were authenticated by the vendors. The cell lines were obtained between 2013 and 2016. The newly received cells were expanded and aliquots of less than 10 passages were stored in liquid nitrogen. All cell lines were kept at low passage, returning to original frozen stocks every 6 months. During the course of this study, cells were thawed and passaged within 2 months in each experiment.

All of the cells were cultured in RPMI-1640 medium or Dulbecco’s modified Eagle’s medium (Gibco, Grand island, NY, USA) as recommended by the American Type Culture Collection (Manassas, VA, USA), and supplied with 10% vol/vol heat-inactivated fetal bovine serum (GIBCO/Invitrogen, Carlsbad, CA, USA) with 100 U/mL penicillin and 100 μg/mL streptomycin (GIBCO/Invitrogen, Carlsbad, CA, USA) at 37 °C in a humidified 5% CO_2_ incubator.

For the in vivo study, male athymic BALB/c nu/nu mice (5 weeks old) and male C57BL/6J mice (6 weeks old) were purchased from SLRC Laboratory Animal Co. Ltd. (Shanghai, China) and maintained in our animal facility for 1 week before use. The mice were housed under specific pathogen-free conditions at 24 ± 1°C and 55 ± 5% humidity in a barrier facility with 12 hour light-dark cycles. All of the animal experiments were performed in accordance with the National Institutes of Health Guide for the Care and Use of Laboratory Animals, with the approval of the center for new drug evaluation and research, China Pharmaceutical University (Nanjing, China).

*Chemicals and antibodies*

Melittin, melittin nano-liposomes (melittin modified by 2% poloxamer 188, application number: 200810234946.9) and blank liposomes were offered by Anhui-baichun Pharmaceutical Ltd. (Hefei, China) and stored at room temperature. Melittin and melittin nano-liposomes are produced as a freeze-dried powder, and each bottle contains 0.5 mg melittin. For use, melittin was weighed and dissolved with normal saline. Sorafenib (Bayer, Shanghai, China) was used as a positive drug in the animal experiments. Z-VAD-FMK (Medchem Express, USA) was used as an irreversible broad-spectrum caspase inhibitor. Polyclonal antibodies against Bcl-2 and caspase-3 were purchased from Santa Cruz Biotechnology (Dallas, USA). Monoclonal antibodies against Mcl-1 and Bax were purchased from Abcam (Cambridge, UK). Polyclonal antibodies against cleaved caspase-3 and PARP were purchased from Wanlei Life Sciences (Shenyang, China). Monoclonal antibodies against β-actin were purchased from proteintech (USA).

*MTT assay*

Hepatic carcinoma cells (Bel-7402, BMMC-7721, HepG2, Hepa 1-6, LM-3, and L02 cells) were seeded in triplicate wells into a 96-well plate (1×10^4^ cells/well/100 μl) and allowed to adhere for 4 hours. The supernatant was removed, and the cells were incubated with medium containing various concentrations of melittin nano-liposomes for 72 hours with untreated cells used as a control. Then, the cells were washed twice with PBS and 3-(4,5-dimethyl-2-yl)-2,5-diphenyltetrazolium bromide solution (5 mg/mL) was added to each well. After 4 hours of incubation, the supernatants in each well were removed and 100 μL of dimethyl sulfoxide was added to solubilize the formazan salt. Ten minutes later, the optical density of the wells was read at 570 nm on a microplate reader (Thermo Fisher Scientific, USA). The half maximal inhibitory concentration (IC50) was calculated according to the proliferation inhibition ratio of the cells.

*Cell apoptosis analysis*

For the apoptosis assay, HepG2 cells were seeded in 6-well plates and treated with melittin nano-liposomes and blank liposomes at 2 μM for 24 hours. Then, the FITC-Annexin V apoptosis assay kit (Miltenyi Biotec, Germany) was used according to the manufacturer’s protocol. After staining, cells were observed by fluorescence microscopy (Leica, Germany) or analyzed by flow cytometry (Merck Millipore Flow Sight, Germany). To observe the morphological change of the cell nucleus, Bel-7402 and SMMC-7721 cells were seeded on sterile cover slips in 6-well plates and also treated with 1 μM melittin nano-liposomes. After 24 hours, cells on the slips were fixed with paraformaldehyde and then stained with 4,6-diamidino-2-phenylindole (DAPI, Sigma, USA) for 5 minutes. Nuclear morphology was evaluated by laser scanning confocal microscopy (Leica, Germany).

*Western Blotting*

HepG2 cells were treated with vehicle, blank liposomes (2 μM), melittin (2 μM) or melittin nano-liposomes (2 μM) for 24 hours. Whole cell extracts were prepared and the concentration of the samples was determined using a bicinchoninic acid protein assay kit according to the manufacturer’s instructions (Thermo Fisher Scientific, USA). Equal 30-μg total protein samples were subjected to electrophoresis on 10% SDS-polyacrylamide gels. Gels were run at 120 V for 0.5-1.5 hours and subsequently transferred to a PVDF membrane (Millipore, Billerica, MA). After blocking nonspecific protein binding, the membranes were incubated with primary antibodies overnight at 4 °C. The secondary antibodies were conjugated to horseradish peroxidase (Santa Cruz Biotechnology Inc., Dallas, USA). Detection of specific proteins was performed with an ECL western blotting kit (ECL-plus, Thermo Scientific) according to the manufacturer’s protocol, and the fluorescence signals were recorded by a luminescent image analyzer (Chemi-Smart 2000, Bio-Rad, USA).

*Terminal deoxynucleotidyl transferase-mediated dUTP nick end-labeling (TUNEL) assay*

Apoptotic cells of liver tissues were investigated using a TUNEL assay kit (Roche Diagnostics GmbH, Mannheim, Germany) according to the manufacturer’s instructions. Photographs were taken with a DMI3000B fluorescence microscope (Leica, Germany).

*Mice HCC tumor models*

Male balb/c nu-nu mice (5 weeks old) and C57BL/6J mice (6 weeks old) and ICR mice (5 weeks old) were used for the animal studies. For the subcutaneous xenograft tumor model, HepG2 or Bel-7402 cells (5×10^6^ cells/mL) in serum free media (200 μl) were injected into the right flank of nude mice. When the tumor was approximately 100 mm^3^, the mice were randomly divided into 6 groups including solvent control group (normal saline), blank liposomes group (8 mg/kg), melittin nano-liposomes groups (2 mg/kg, 4 mg/kg and 8 mg/kg) and positive drug group (sorafenib tosylate, 30 mg/kg). Each group contained 8 mice, and the drugs were given intravenously except sorafenib, which had an intragastric administration. The dose and route of melittin and sorafenib was determined according to the clinical application of the drugs. Tumor sizes were measured using a sliding caliper twice a week, and the experiment was terminated when the average tumor volume exceeded 2000 mm^3^. The tumor volume was calculated using the following formula: V (mm^3^) = (length × width^2^) / 2.

Two orthotopic transplantation tumor models were performed as follows. Mice were anesthetized with 4% chloral hydrate (0.1 mL/10 g) and placed in a supine position. A small transverse incision below the sternum was made to expose the liver. Hepa 1-6 or LM-3-GFP cells (1×10^6^ cells) were suspended in 20 μl PBS, which was mixed with 50% Matrigel (BD Biosciences) and injected into the upper left lobe liver capsule of C57BL/6J mice or Balb/c nu-nu mice. Then, the abdomen was sutured. Ten days after injection, the mice were randomly divided into 6 groups, including solvent control group (normal saline), blank liposomes (8 mg/kg), melittin nano-liposome groups (2 mg/kg, 4 mg/kg and 8 mg/kg) and positive drug group (Sorafenib tosylate, 30 mg/kg). The experiment was terminated when death occurred. Antitumor efficacy was assessed by measuring the tumor directly or detecting the fluorescence intensity of GFP using an in-vivo imaging system (Eastman Kodak Company, Rochester, USA).

To evaluate the toxicity of melittin and melittin nano-liposomes, ICR mice were randomly divided into 4 groups, according to their body weight, with 10 mice each: normal saline, blank liposomes (2 mg/kg), melittin (2 mg/kg) and melittin nano-liposomes (2 mg/kg) groups. Drugs were administered once daily for two weeks. At the endpoint, peripheral blood was collected and subsequently analyzed by an automated hematology analyzer (Siemens ADVIA 2120 Hematology System). Livers were treated with formalin and processed into paraffin sections for the TUNEL assay and spleens were collected and grinded into a cell suspension for further splenic immune cell analysis by flow cytometry.

*Flow cytometric analysis*

Peritoneal macrophages and spleens were harvested from the experimental mice. To obtain mouse peritoneal macrophages, the peritoneal cavity was gently flushed with 5 mL of sterile PBS and harvested. Spleens were homogenized, passed through a 70 mm nylon sieve and resuspended in PBS to obtain a cell suspension. Pretreatment with blocking antibodies was performed with anti-mouse CD45-BV510, CD4-APC, CD8-PECy7, CD11b-PE, Ly6G-PECy7, F4/80-FITC, or CD19-PECy5 (eBioscience, California, USA) according to the manufacturer’s instructions. Samples were detected by flow cytometry (Merck Millipore Flow Sight, Germany).

*Statistical analysis and experiment repeat*

Results were calculated from three independent experiments and plotted as SEM. Statistical significance was calculated using the Anova Two-Way test (*p ≤ 0.05; **p ≤ 0.01; ***p ≤ 0.001). All of the in vitro experiments were performed three times. At least 5 animals were used for in vivo experiments.

**References**

1. Eisenhauer EA, Therasse P, Bogaerts J, Schwartz LH, Sargent D, Ford R, et al. New response evaluation criteria in solid tumours: revised RECIST guideline (version 1.1). Eur J Cancer 2009; 45:228-47.
