# Supplementary material for: A novel melittin nano-liposome exerted excellent anti-hepatocellular carcinoma efficacy with better biological safety
Source: J Hematol Oncol. 2017 Mar 20;10:71. doi: 10.1186/s13045-017-0442-y (PMC5359812; doi:10.1186/s13045-017-0442-y)

a

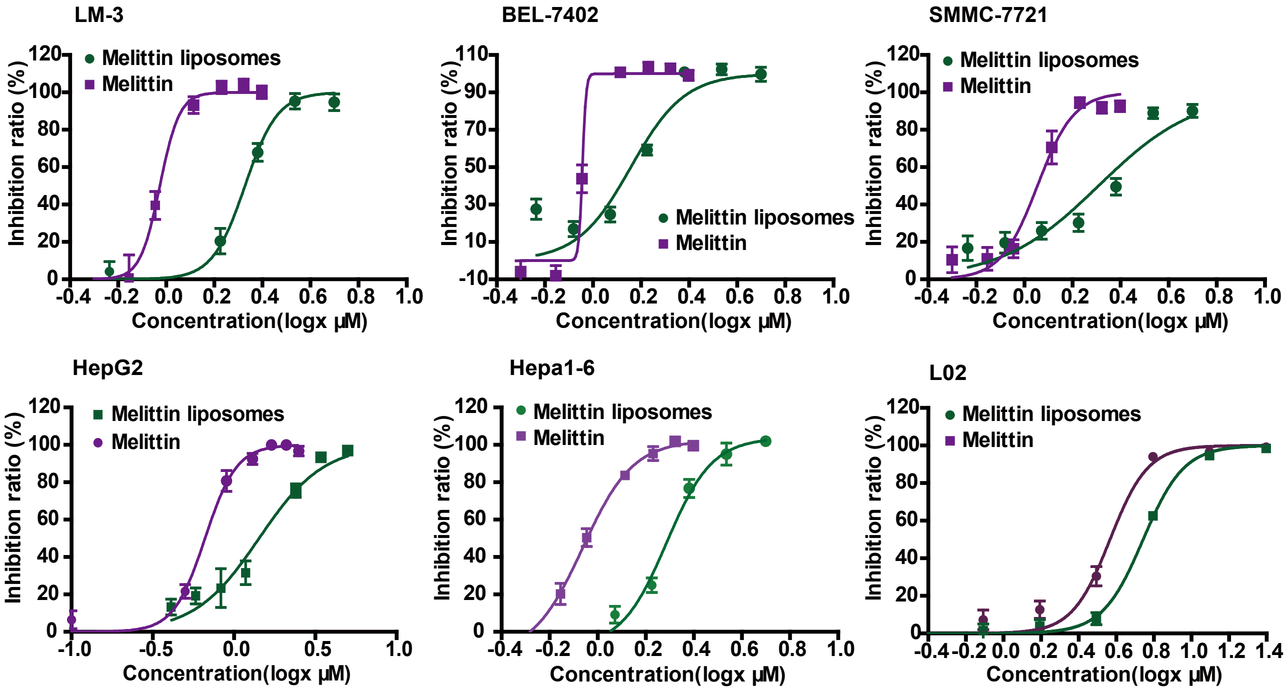

Cell lines	Mel (μM)	Mel-lipo (μM)
LM-3	0.94	2.13
Bel-7402	0.90	1.44
SMMC-7721	1.13	2.03
HepG2	0.66	2.19
Hepa 1-6	0.87	1.93
L02	3.69	5.54

b

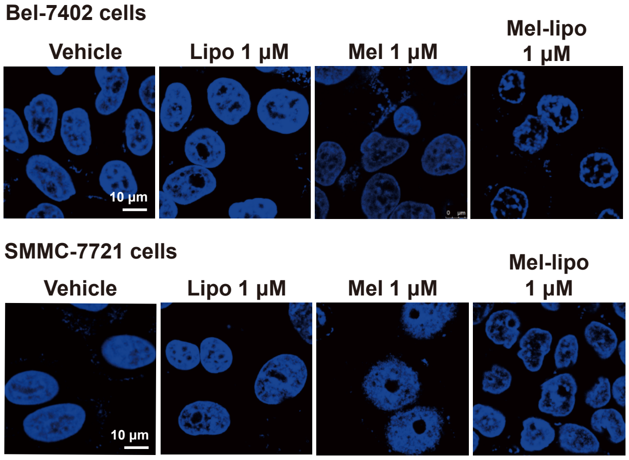

c HepG2 cells

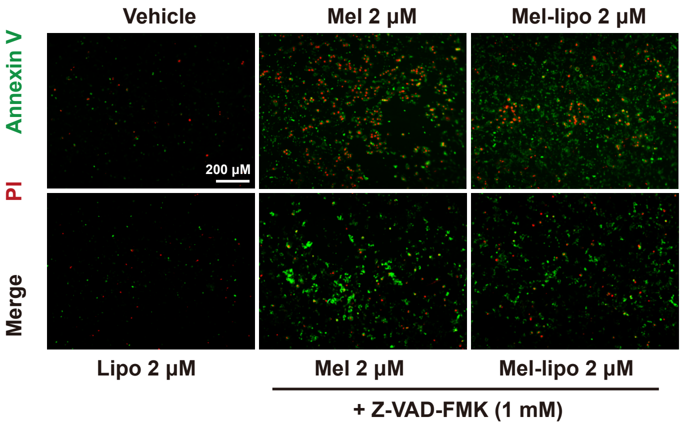

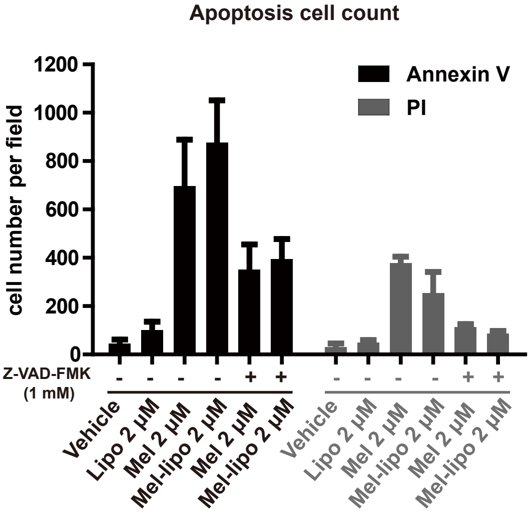

Supplement: Additional file 2: Figure S1. — The effects of melittin nano-liposomes on the proliferation and apoptosis of tumor cells. (a) Proliferation inhibiting rates of melittin and melittin nano-liposomes on various HCC cells lines including LM-3, Bel-7402, SMMC-7721, HepG2, and L02 cells. (b) Cell nucleus staining by DAPI to observe the apoptosis of Bel-7402 and SMMC-7721 cells after treated with vehicle, blank liposomes (1 μM), melittin (1 μM), or melittin nano-liposomes (1 μM) for 24 h. (c) Fluorescence staining of Annexin V-FITC and PI to detect the apoptosis of HepG2 cells after treatment with melittin and Melittin nano-liposomes for 24 h and observed by fluorescence microscope. HepG2 cells were pretreated with Z-VAD-FMK for 6 hours, and melittin and Melittin nano-liposomes were subsequently administered at a concentration of 2 μM. (PDF 848 kb) [file 13045_2017_442_MOESM2_ESM.pdf]
