# Supplementary figures and images for: A novel melittin nano-liposome exerted excellent anti-hepatocellular carcinoma efficacy with better biological safety
Source: J Hematol Oncol. 2017 Mar 20;10:71. doi: 10.1186/s13045-017-0442-y (PMC5359812; doi:10.1186/s13045-017-0442-y)

Subcutaneous transplanted tumors of HepG2 cells

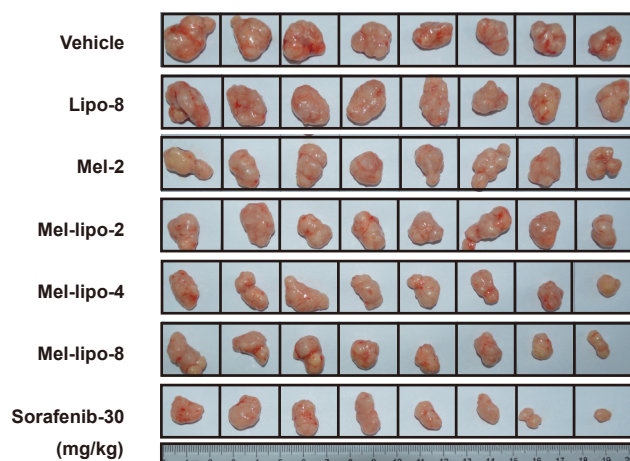

Tumor weight of HepG2 model

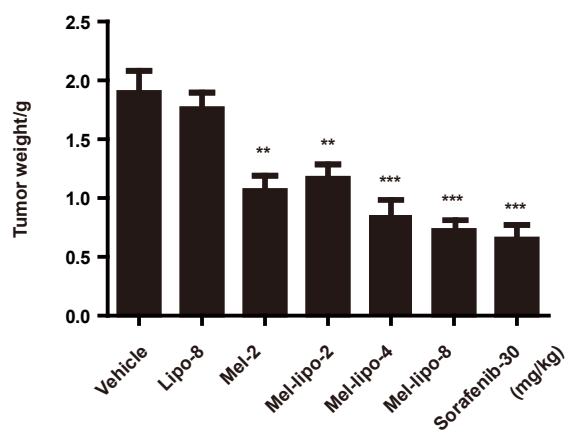

Supplement: Additional file 3: Figure S2. — Photo of HepG2 tumors of vehicle, blank liposomes (8 mg/kg), melittin (2 mg/kg), melittin nano-liposomes (2, 4, and 8 mg/kg), and sorafenib (30 mg/kg) treated groups in the HepG2 subcutaneous transplanted tumor model. The data are presented as the mean ± SEM. Statistical significance was calculated using Student’s t test (**p ≤ 0.01; ***p ≤ 0.001). (PDF 1070 kb) [file 13045_2017_442_MOESM3_ESM.pdf]

### Splenic T lymphocytes

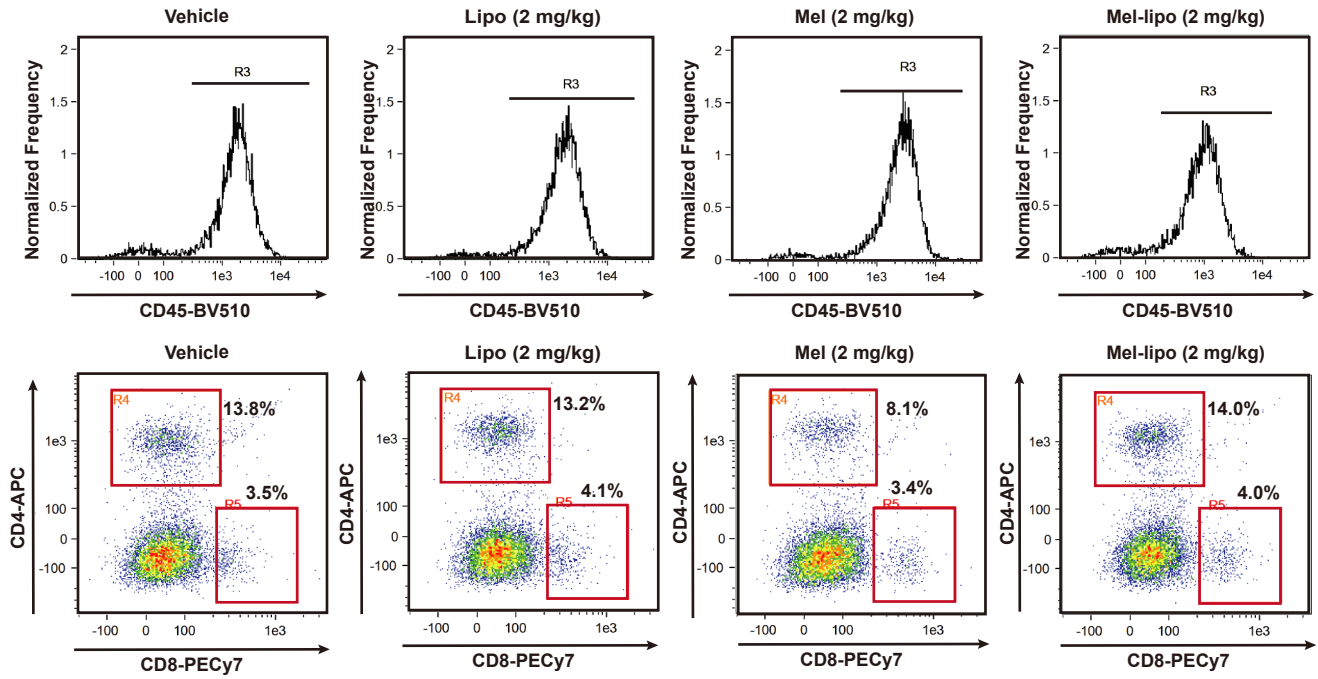

### Splenic neutrophil

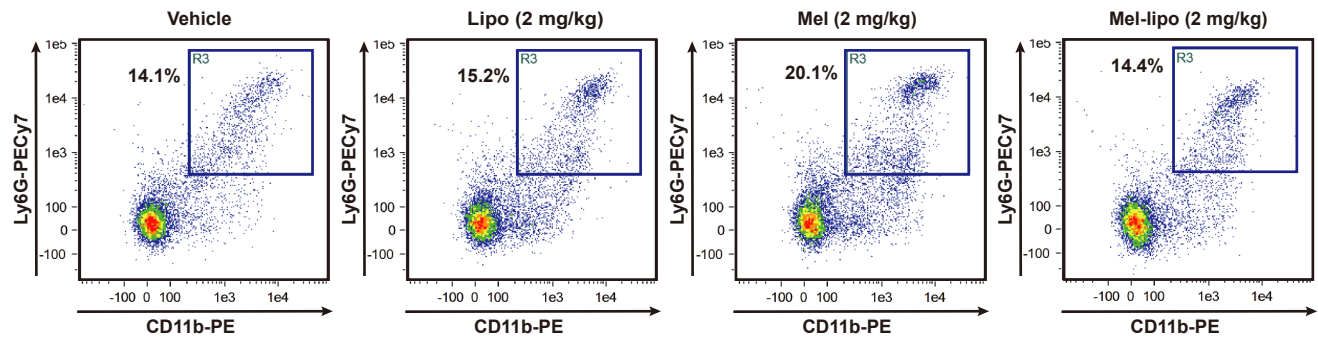

### Splenic B lymphocytes

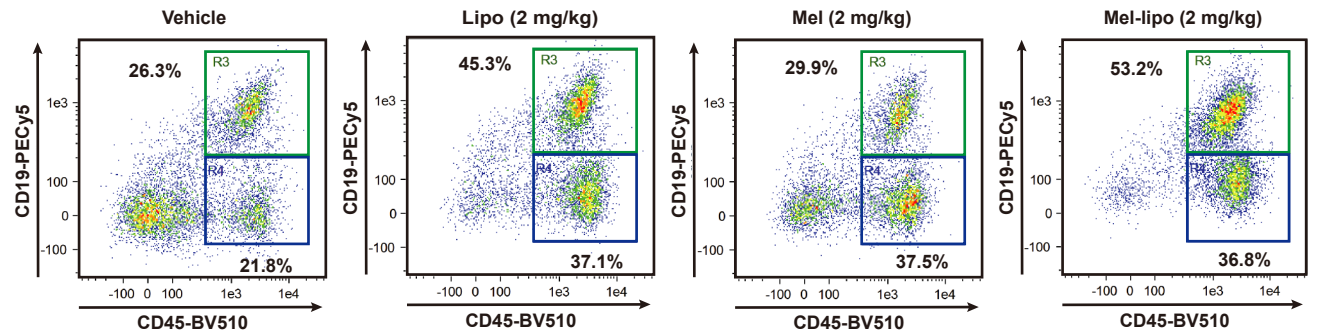

Supplement: Additional file 4: Figure S3. — Flow cytometry analysis of splenic immune cells including splenic T lymphocytes, neutrophil and B lymphocytes. Spleens were stripped and grinded into cell suspension after mice were treated with vehicle, blank liposomes (8 mg/kg), melittin (2 mg/kg) and melittin nano-liposomes (2 mg/kg) for two weeks. (PDF 730 kb) [file 13045_2017_442_MOESM4_ESM.pdf]
